# Taurine detected using high-resolution magic angle spinning ^1^H nuclear magnetic resonance: A potential indicator of early myocardial infarction

**DOI:** 10.3892/etm.2012.857

**Published:** 2012-12-13

**Authors:** YUNLONG YANG, LIN YANG, YUE ZHANG, XINGHUA GU, DANLING XU, FANG FANG, AIJUN SUN, KEQIANG WANG, YIHUA YU, JI ZUO, JUNBO GE

**Affiliations:** 1 Zhongshan Hospital of Fudan University, Shanghai Institute of Cardiovascular Disease, Shanghai 200032;; 2 Department of Cellular & Genetic Medicine, Shanghai Medical School, Fudan University, Shanghai 200032;; 3 Research Center, Eye & ENT Hospital of Fudan University, Shanghai 200031;; 4 Shanghai Key Laboratory of Functional Magnetic Resonance Imaging, Physics Department, East China Normal University, Shanghai 200062, P.R. China

**Keywords:** taurine, high-resolution magic angle spinning, myocardial infarction, high-performance liquid chromatography

## Abstract

Magnetic resonance spectroscopy (MRS) is a unique non-invasive method for detecting cardiac metabolic changes. However, MRS in cardiac diagnosis is limited due to insensitivity and low efficiency. Taurine (Tau) is the most abundant free amino acid in the myocardium. We hypothesized that Tau levels may indicate myocardial ischemia and early infarction. Sprague-Dawley rats were divided into seven groups according to different time points during the course of myocardial ischemia, which was induced by left anterior descending coronary artery ligation. Infarcted myocardial tissue was obtained for high-resolution magic angle spinning ^1^H nuclear magnetic resonance (NMR) analysis. Results were validated via high-performance liquid chromatography. The Tau levels in the ischemic myocardial tissue were reduced significantly within 5 min compared with those in the control group (relative ratio from 20.27±6.48 to 8.81±0.04, P<0.05) and were maintained for 6 h post-ischemia. Tau levels declined more markedly (56.5%) than creatine levels (48.5%) at 5 min after ligation. This suggests that Tau may have potential as an indicator in the early detection of myocardial ischemia by ^1^H MRS.

## Introduction

Cardiovascular diseases (CVD) are a significant cause of mortality worldwide and the main cause of CVD is acute myocardial infarction (AMI). In the United States, acute coronary syndrome (ACS) affects 1.3 million individuals (1). Clinically, early detection is essential for the treatment and prognosis of myocardial infarction. At present, the serum levels of cardiac troponins cTnI and cTnT, are used to diagnose early myocardial infarction (2). In addition, the uptake of radioactive tracers, such as exogenous glucose and acetate, is applied clinically to predict cardiac functional improvement (3).

Ischemia and myocardial infarction are different stages in the progression of heart failure. Identifying the early changes of various metabolic parameters in myocardial ischemia is of clinical importance (4). The myocardium benefits from reperfusion-based therapeutic approaches if ischemia is detected in the initial period. Therefore, detecting early metabolic parameter changes in myocardial ischemia may have diagnostic value (5).

Magnetic resonance spectroscopy (MRS) is a unique non-invasive method for detecting cardiac metabolic changes, which usually occur significantly earlier than irreversible organic lesions in myocardial infarction (6). This method detects biochemical processes and evaluates metabolism without blood sampling or the use of radionuclides (5). To date, cardiovascular magnetic resonance studies using ^1^H MRS have focused on its sensitivity and specificity in the study of myocardial ischemia (7,8).

^31^P-MRS is commonly used to study myocardial energy metabolism (9,10). In previous studies, ^31^P-MRS has been employed to measure indicators, including phosphocreatine (PCr)/ATP and PCr/inorganic phosphate (Pi) ratios, in humans to guide heart transplantation and myocardial infarction non-invasively (6,11,12).

It is well recognized that protons have the highest magnetic moment of all biologically relevant nuclei, with a high concentration in organic molecules. These features make it possible to achieve enhanced spatial resolution by ^1^H MRS. Compared with ^31^P MRS, ^1^H MRS provides higher sensitivity and detects more metabolites, such as unphosphorylated creatine (3,13). ^1^H nuclear magnetic resonance (NMR) has been used to evaluate heart tissue *ex vivo* to produce a high-resolution metabolic profile in myocardial infarction animal models. However, cardiac motion, respiratory motion and epicardial fat make it difficult to apply ^1^H NMR clinically (5).

Metabolomics is a rapidly evolving field that aims to identify and quantify the concentration changes of all metabolites in a model system. This approach involves large metabolite datasets and high-throughput techniques, including NMR spectroscopy or mass spectroscopy. High-resolution magic angle spinning (HRMAS) ^1^H NMR spectroscopy has been used to investigate cardiac metabolites in rodent models (14–17). Multivariate pattern recognition analysis coupled with the use of HRMAS was able to identify metabolic biomarkers of disease in intact tissue. The metabolic information obtained from HRMAS spectra may be transferred to the clinical environment (18,19).

Taurine (Tau), a sulfur-containing β-amino acid, is the most abundant free amino acid in the myocardium (∼60%) (4). The Tau content is high in mammalian hearts, ranging between 5 and 40 *μ*mol/g wet weight (20). It has been demonstrated that Tau has a number of functions, including the regulation of intracellular calcium balance, antioxidant and anti-inflammatory actions, immune regulation and cardiovascular protection (4,20–22). Cardiac muscle lacks the ability to synthesize Tau (23). This suggests that Tau originates from a transport process. It has been recognized that myocardial Tau synthesis is limited while the majority of the Tau in cardiac tissue is accumulated by uptake from the blood (20). As such, myocardial Tau levels may change when ischemia occurs. The Tau transporter, located on the cell membrane, is important in Tau metabolism in the myocardium. In Tau transporter knockout mice, the level of Tau was decreased by 98% in the myocardium, which indicates that the Tau uptake process is solely Tau transporter dependent without compensation from other transport systems (24,25). We hypothesize that changes in the Tau level in myocardial tissue may be a potential indicator of myocardial ischemia and early infarction.

In the present study, a myocardial ischemia model was created in rats. Metabolic markers, including Tau, creatine (Cre), choline (Cho) and lactate (Lac), were analyzed at various time points *ex vivo* using HRMAS ^1^H NMR. The results were further confirmed by high performance liquid chromatography (HPLC).

## Materials and methods

### Animal model and cell culture

The animals were provided by the Animal Center of Fudan University (Shanghai, China). All procedures were performed with approval from the Animal Care and Use Committee of Shanghai, China. Adult Sprague-Dawley (SD) rats (male, body weight 200–250 g) were anesthetized with pentobarbital sodium (30 mg/kg I.P.; Sigma, St. Louis, MO, USA; Lot No. P3761). The left anterior descending coronary artery (LAD) was ligated with a 6-0 suture (Jinhuan Medical, Shanghai, China; Lot No. 20F101) as previously reported (26). Sham-operated animals underwent the same procedure but the LAD was left untied. The animals were divided into seven groups according to the time points following ligation: 0 min (control, sham-operated), 5 min, 20 min, 30 min, 45 min, 1 h and 6 h. The heart was then excised. The infarction zone was isolated and then frozen in liquid nitrogen for further analysis. For three rats from the 6 h group, the rat LAD ligation model was verified by 1% triphenyltetrazolium chloride (TTC) and Evans Blue double staining, as previously described (27). The H9c2 rat cardiomyoblast cell line was obtained from the Cell Bank of the Chinese Academy of Sciences (Beijing, China). For the HPLC and apoptosis assay, the cells were cultured in Dulbecco’s modified Eagle’s medium (DMEM, HyClone, Waltham, MA, USA) supplemented with 10% FBS, 100 U/ml penicillin G and 100 mg/ml streptomycin. The cells were cultured in standardized cell culture incubator conditions at 37°C in a humidified atmosphere containing 5% CO_2_.

### HRMAS ^1^H NMR

For the HRMAS ^1^H NMR analysis, the tissue samples (from the control and myocardial ischemia groups, including the 5 min, 20 min, 30 min, 45 min, 1 h and 6 h groups, n≥3), weighing 25±2 mg each, were placed into a 25 *μ*l zirconium oxide rotor with drops of D_2_O (deuterium lock reference). NMR tests were performed on a Bruker DRX-500 (Bruker BioSpin GmbH, Rheinstetten, Germany) spectrometer (^1^H frequency at 500.13 MHz) at 300.0 K, with a rotor spin rate of 5 kHz. Carr Purcell Meiboom Gill (CPMG) pulse sequences were used with solvent presaturation during the relaxation delay of 2 sec. The NMR spectra were acquired with 256 scans collected into 64,000 data points with a spectral width of 15 kHz. The CPMG pulse sequence was applied to suppress signals from the molecules with short T2 values, such as macromolecules and lipids, using a total echo time (TE) of 320 msec. The stability of tissue samples was evaluated by repeating a one-dimensional NMR experiment following overall acquisition. No biochemical degradation was observed in any of the tissue samples. Spectral assignments were further confirmed by two-dimensional ^1^H-^1^H total correlation spectroscopy (TOCSY)and ^1^H-^1^H correlation spectroscopy (COSY; data not shown) with values obtained from the literature (14,28).

### Principal component analysis (PCA)

Spectral data were phased and baseline-corrected using XWINNMR (Bruker Biospin GmbH). All free induction decays (FIDs) were multiplied by an exponential function equivalent to a 0.3-Hz line-broadening factor prior to Fourier transformation. Each HRMAS ^1^H NMR spectrum was segmented into 236 regions of equal width (0.04 ppm) over the region δ0.00–10.00 and the signal intensity in each region was integrated by AMIX (version 3.6, Bruker Biospin GmbH). The region δ4.40–5.00 was removed to eliminate the baseline effects of imperfect water saturation. Prior to PCA, each integral region was normalized by dividing by the sum of all integral regions for each spectrum (29,30). PCA was used to calculate a new, smaller set of orthogonal variables from linear combinations of the intensity variables while retaining the maximum variability present within the data. These new variables were the derived principal components and the distribution of their values (scores) permitted the simple visualization of separation or clustering between samples. The weightings (loadings) applied to each integral region in calculating the principal components allowed for the identification of those spectral regions having the greatest effects on separation and clustering and, hence, the deduction of the characteristic metabolites of myocardial ischemia.

### HPLC

The ischemic myocardial tissue (250 mg) was homogenized by ultrasound (BILON96-II; Bilon Instruments, Shanghai, China) and mixed with 18% sulfosalicylic acid (SCRC, 250 *μ*l/100 mg). The mixture was centrifuged at 13,000 rpm for 5 min. The supernatant was then taken and filtered through a 0.22 *μ*m membrane. O-phthalaldehyde (OPA) precolumn derivatization was performed as previously described (31). After OPA derivatization, the sample extract was immediately detected by HPLC (Agilent 1100; Agilent, Santa Clara, CA, USA) with the parameters as follows: column, ZORBAX Eclipse XDB-C18 4.6x150 mm, 5 *μ*m (Agilent); mobile phase A, methanol:acetonitrile:H_2_O = 45:45:10 (v/v/v); mobile phase B1, methanol (0.05 mol/l):sodium acetate buffer (pH 5.3):tetrahydrofuran = 42:57:1; mobile phase B2, 40 mM phosphate buffer (Na_2_HPO_4_, pH 7.8). Fluorescence detection was performed at 450 nm. L-norvaline (Agilent, Lot No.1103756) was added as the internal standard.

### Hypoxia treatment and apoptosis assay

A cardiac myoblast cell line hypoxia model was established as described previously (32). Briefly, after washing with PBS, the cells were placed in serum- and glucose-free DMEM and incubated in a sealed, hypoxic anaerobic rectangular jar fitted with a catalyst (BioMérieux, Marcy l’Etoile, France) to scavenge free oxygen. For the Tau treated group, Tau was added to these cultures (final concentration 40 nM) and allowed to incubate for 3 h before hypoxia treatment was commenced. The myocardial cells were digested and collected by centrifuging. The myocardial cell single cell suspension was stained with an apoptosis assay kit (Annexin V-FITC and propidium iodide, KeyGene Biotech, Wageningen, the Netherlands) at room temperature for 15 min. The sample was then evaluated by flow cytometry (BD FACS Calibur) and analyzed using CellQuest (BD, version 5.1).

### Statistical analysis

Statistical analysis was performed using the SPSS statistical program (version 11.0, SPSS Inc., Chicago, IL, USA). All values were expressed as the mean ± standard deviation (SD). Differences between groups were evaluated using a one-factor ANOVA test and P<0.05 was considered to indicate a statistically significant difference.

## Results

### LAD ligation model and HRMAS ^1^H NMR spectroscopy

**Figure 1.**
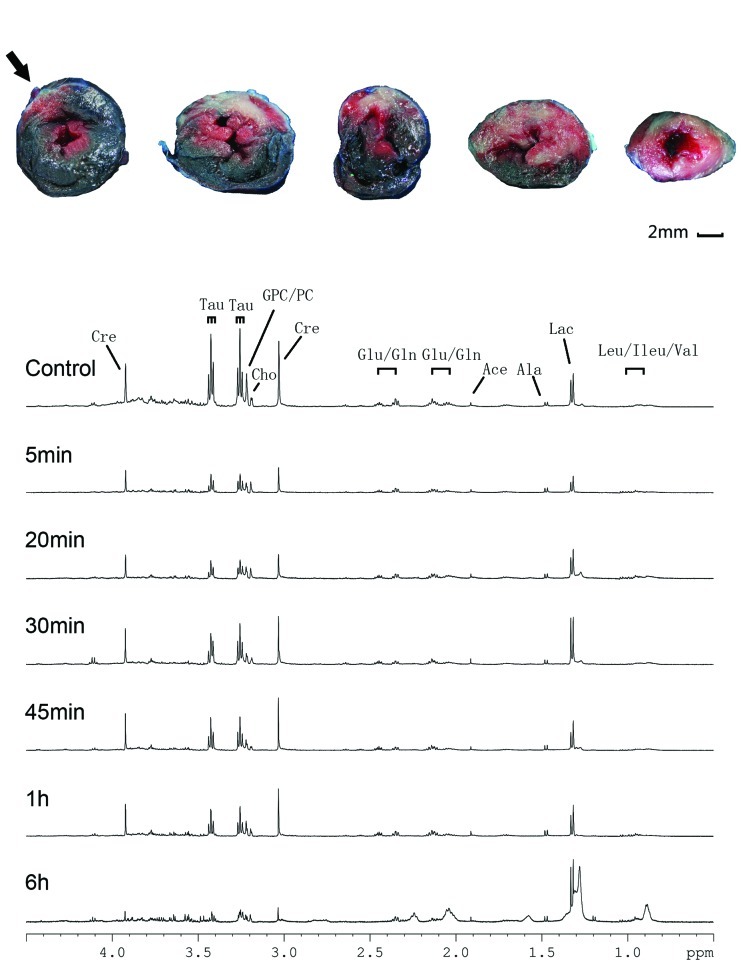
TTC/Evans Blue staining and HRMAS ^1^H CPMG NMR spectra of the infarcted myocardium at various time points. (A) Five rat heart slices from the anatomical top to bottom are shown from left to right; infarct area (white), risk area (unstained) and non-ischemic area (blue) are shown and the ligation site is indicated by an arrow (bar = 2 mm). (B) Peak assignments are as follows: Cre, creatine; Tau, taurine; GPC/PC, glyceryl phosphorylcholine/phosphorylcholine; Cho, choline; Glu/Gln, glutamate/glutamine; Ace, acetone; Ala, alanine; Lac, lactate; and Leu/Ileu/Val, leucine/isoleucine/valine. TTC, triphenyltetrazolium chloride; HRMAS, high-resolution magic angle spinning; CPMG, Carr Purcell Meiboom Gill; NMR, nuclear magnetic resonance.

TTC and Evans Blue double staining were performed on excised hearts obtained from the 6 h group. The infarct area (TTC unstained), risk area (Evans Blue unstained) and non-ischemic area (Evans Blue stained) are shown in Fig. 1A. The result indicated that the LAD ligation model effectively mimicked myocardial infarction.

Representative ^1^H CPMG NMR spectra of the control, 5 min, 20 min, 30 min, 45 min, 1 h and 6 h groups following LAD ligation are shown in Fig. 1B. The main metabolites, including leucine (Leu), isoleucine (Ileu), valine (Val), Lac, alanine (Ala), glutamate (Glu), glutamine (Gln), Cre, Cho, phosphorylcholine (PC), glyceryl phosphorylcholine (GPC) and Tau were assigned. The Leu/Ileu/Val and Lac levels increased following LAD ligation, whereas the Tau, Cho and PC/GPC levels decreased in a time dependent manner compared with those in the control group,.

### Tau decreased most notably among the potential markers ex vivo

**Figure 2.**
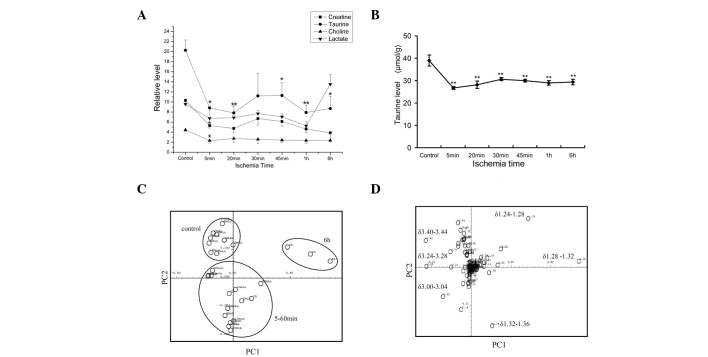
Taurine decreased most notably of the potential markers *ex vivo*. (A) Levels of creatine, taurine, choline and lactate over ischemia time; the y-axis shows the relative level adjusted by alanine level; all statistical significances were tested in comparison with the control. ^*^P<0.05, ^**^P<0.01 vs. 0 min, n≥3. (B) Taurine was derivatized by OPA and detected by HPLC; L-norvaline was used as the inner control; conditions as described in Materials and methods; ^*^P<0.05, ^**^P< 0.01 vs. 0 min, n≥3 (C) Score plot of the first (PC1) and second (PC2) principal components (horizontal and vertical axis, respectively) of the ^1^H NMR spectra. Samples were divided into three groups: a control group, a 5–60 min group and a 6 h group. (D) The corresponding loading plot of the PC1 and second PC2 principal components (horizontal and vertical axis, respectively) of the ^1^H NMR spectra. Bands contributing the most to the distinction between the groups are shown. OPA, O-phthalaldehyde; HPLC, high-performance liquid chromatography; NMR, nuclear magnetic resonance.

The data acquired from the ^1^H CPMG NMR spectra were analyzed and metabolites were assigned. The levels of potential markers, including Tau, Cre and Cho, are shown in Fig. 2A. The Tau, Cre and Cho levels declined significantly in the 5 min ischemia group compared with the controls (P<0.05). In the 20 min to 6 h groups, Tau, Cre and Cho remained at low levels, while Lac accumulated from 1 to 6 h. At 5 min after ischemia, the relative Tau level decreased from 20.27±6.48 to 8.81±0.04 (56.5%); this reduction was greater than that of Cre (48.5%) or Cho (47.7%).

PCA was applied to all the CPMG ^1^H NMR spectra results (Fig. 2C and D). In the score plot (Fig. 2C), the control group showed intra-group similarity. Samples from the 5 min to 1 h groups clustered together, while the 6 h group formed another cluster. The three clusters were separated from each other.

The corresponding loading plot (Fig. 2D) revealed that lipids (δ1.24–1.28 and δ1.28–1.32), Lac (δ1.32–1.36), Cr (δ3.00–3.04), PC/GPC (δ3.24–3.28) and Tau (δ3.24–3.28 and δ3.40–3.44) contributed the most to the separation. The lipids mainly contributed to the identification of the 6 h group. In the area of small molecule metabolites (δ3.00–3.44), Tau contributed the most to distinguishing the ischemia group from the control group. The PCA result was in accordance with the CPMG ^1^H NMR spectral results.

The myocardial tissue Tau level was confirmed by HPLC (Fig. 2B). Consistent with the results of the spectroscopy, the Tau level decreased significantly within 5 min when compared with the control group (P<0.01). No further changes were observed after 5 min.

These results revealed that, within potential metabolic markers, Tau experienced the largest reduction in its levels within the first 5 min. Tau contributed the most to distinguishing the ischemia group from the control group. The NMR *ex vivo* detection was confirmed by HPLC.

### Tau decreases in vitro and shows protective effects in hypoxia

**Figure 3.**
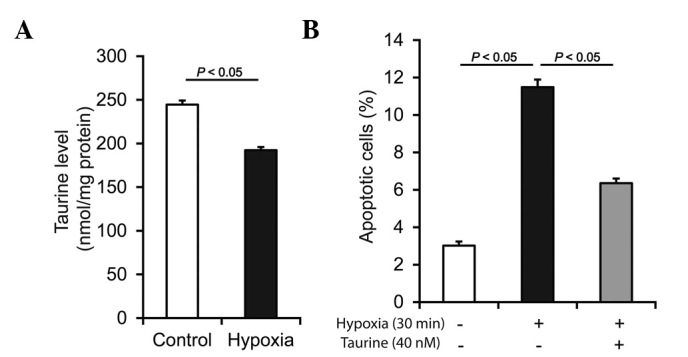
Taurine decreased *in vitro* and showed a protective effect in hypoxia induced apoptosis. (A) Summary of the quantified taurine levels in the H9c2 cell line, demonstrating a significantly decreased taurine level following hypoxia injury. ^*^P<0.05, n≥3. (B) Summary of the apoptosis assay in the H9c2 cell line, demonstrating a significant increase in apoptotic cell number following hypoxia injury, while taurine treatment significantly reduced the number of apoptotic cells. ^*^P<0.05, n≥3.

To confirm the findings, the Tau level was detected *in vitro*. Myocardial infarction may be described as oxygen and glucose deprivation. As shown in Fig. 3A, following hypoxia injury, the Tau level decreased significantly in the H9c2 rat cardiomyoblast cell line. In accordance with the *ex vivo* data, the results showed that the Tau level also decreased *in vitro*. Moreover, hypoxia increased H9c2 cell apoptosis *in vitro*, while Tau demonstrated a protective effect against hypoxia-induced myocardial cell apoptosis (Fig. 3B). This result suggests that, besides being a potential indicator of ischemia, Tau may also have therapeutic potential, which is of greater clinical importance.

## Discussion

The HRMAS ^1^H NMR technique is a developing technique in NMR spectroscopy. This approach requires minimal sample preparation and, unlike conventional spectroscopy of tissue extracts, enables aqueous and lipid-soluble metabolites to be observed simultaneously *in situ*. Therefore, HRMAS ^1^H NMR is an efficient method for studying various tissue abnormalities.

Tau is a sulfur-containing β-amino acid present at a high concentration in the myocardium. It has been widely used as a nutritional supplement and therapeutic tool for cardiovascular diseases, including congestive heart failure, myocardial infarction and hypertension (20,21).

In the present study, ischemic myocardial tissue samples from various time points were used for HRMAS ^1^H NMR detection. It has been reported that Cre levels decreased significantly following LAD ligation in a swine model (5). The present study showed that in the rat LAD ligation model, Tau and Cre levels decreased significantly within 5 min compared with those in the control group. This result indicates that Tau may be an indicator of early heart ischemia as well as Cre. Cardiac tissue lacks a Tau synthesis mechanism and the myocardial Tau level is Tau transporter-dependent. Therefore, the rapid decline of Tau levels may reflect myocardial ischemia.

At present, troponin (troponins T, I and C) is used as the golden standard in the clinical diagnosis of myocardial infarction. Troponins T and I have distinct isoforms that exist in skeletal and cardiac muscle. The release of these proteins from necrotic cardiomyocytes into the bloodstream accounts for their utility as biomarkers of acute coronary syndromes (33). However, irreversible changes in the myocardium occur at 6 h postischemia, while troponin levels increase at 4–8 h postischemia and reach a peak at 12–48 h (34). The levels of a novel diagnostic indicator, heart type fatty acid binding protein (H-FABP), increase at 1–3 h postischemia (35,36). However, the change in the Tau level may be detected in the ischemic zone at 5 min after LAD ligation, which is significantly earlier compared with the detection of increased troponin or H-FABP levels. With regard to specificity, troponin exists solely in the myocardium, while Tau is present at high levels in the myocardium and skeletal muscle. However, MRS detection would be able to focus on myocardial tissue *in vivo* specifically and non-invasively if improved algorithms were developed to eliminate cardiac and respiratory motion. In the present *ex vivo* study, ischemic tissue was excised and detected by HRMAS ^1^H NMR. Further investigation of Tau detection *in vivo* is expected.

It has previously been shown that Cre levels, belonging to the myocardial energy index, are related to myocardial functional performance under ischemic conditions in animal hearts *in vivo*(37). In ^31^P-MRS, PCr and ATP represent the level of myocardial energy. A reduced PCr/ATP ratio indicates cardiac failure and is a predictor of mortality. A decrease in total Cre is an early signal of heart failure (38). Total Cre may serve as a compensatory mechanism for minimizing the reduction of the total purine pool in the failing heart and reflects changes in myocardial function (12). In acute myocardial ischemia, which may lead to heart failure (39), energy loss appears significantly earlier than functional changes. The present data showed that Tau and Cre levels decreased in the early period of ischemia. The association of Tau with energy consumption in the myocardium merits further investigation.

Clinically, myocardial ischemia may also be detected by MRI (40). However, MRS provides mostly chemical composite information which is useful for research and diagnosis. It is possible that MRI and MRS may interface more often within cardiovascular magnetic resonance studies. Tau may serve as an indicator of early myocardial ischemia. This also raises an interesting question as to whether the Tau metabolism in the heart tissue as well as association of Tau with energy metabolism should also be studied.

In conclusion, by detecting metabolite changes using HRMAS ^1^H NMR, it was observed that Tau levels decreased significantly within 5 min after ischemia in myocardial tissue. These results suggest that the Tau level is a potential indicator of early myocardial ischemia in cardiovascular magnetic resonance studies.
